# Understanding and Embracing Culture in International Faculty Development

**DOI:** 10.5334/pme.31

**Published:** 2023-01-04

**Authors:** Sara Mortaz Hejri, Rashmi Vyas, William P. Burdick, Yvonne Steinert

**Affiliations:** 1 Institute of Health Sciences Education, Faculty of Medicine and Health Sciences, McGill University, Montreal, Canada; 2 FAIMER (The Foundation for Advancement of International Medical Education and Research), member of Intealth, and Director FAIMER Global Programs, FAIMER Philadelphia, USA; 3 The Network: Towards Unity for Health (TUFH) and Founding Co-Director Emeritus of the FAIMER Institute, USA; 4 Family Medicine and Health Sciences Education, the former director of the Institute of Health Sciences Education, and the Richard and Sylvia Cruess Chair in Medical Education, Faculty of Medicine and Health Sciences, McGill University, Montreal, Canada

**Keywords:** Faculty development, culture, cultural diversity, transformative learning theory

## Abstract

**Introduction:**

Research on international faculty development programs (IFDPs) has demonstrated many positive outcomes; however, participants’ cultural backgrounds, beliefs, and behaviors have often been overlooked in these investigations. The goal of this study was to explore the influences of culture on teaching and learning in an IFDP.

**Method:**

Using interpretive description as the qualitative methodology, the authors conducted semi-structured interviews with 15 Fellows and 5 Faculty of a US-based IFDP. The authors iteratively performed a constant comparative analysis to identify similar patterns and themes. Transformative Learning Theory informed the analysis and interpretation of the results.

**Results:**

This research identified three themes related to the influences of culture on teaching and learning. First, cultural differences were not seen as a barrier to learning; instead, they tended to act as a bridge to cultural awareness and network building. Second, some cultural differences produced a sense of unease and uncertainty, which led to adaptations, modifications, or mediation. Third, context mattered, as participants’ perspectives were also influenced by the program culture and their professional backgrounds and experiences.

**Discussion:**

The cultural diversity of health professions educators in an IFDP did not impede learning. A commitment to future action, together with the ability to reflect critically and engage in dialectical discourse, enabled participants to find constructive solutions to subtle challenges. Implications for faculty development included the value of enhanced cultural awareness and respect, explicit communication about norms and expectations, and building on shared professional goals and experiences.

## Introduction

The number of international faculty development programs (IFDPs), designed to promote capacity-building among health professions educators across different countries, is growing [1,2]. Program evaluations have demonstrated increases in participants’ knowledge and skills, and the creation of transnational communities [1,3,4,5,6,7]. However, despite the diversity of program participants’ cultural backgrounds, very few studies [8,9] have explored the influence of participants’ beliefs, values, and behaviors on participation and learning. Educators have also lamented that the notion of “one size fits all” prevails in the faculty development literature [10].

IFDPs refer to two types of programs: those that bring together participants from different countries around the world [3,4,6,7] and those that include the transfer of a faculty development program from one country to another [5,11,12,13,14,15]. A scoping review of 24 reports of IFDPs stated that “although close to 50% of these reports acknowledged the importance of national contexts or cultural norms and beliefs”, cultural issues in IFDPs have not been systematically studied [1]. In addition, a review of primary publications [3,4,5,6,7,12,13,14,15,16] revealed that little is currently known about the types of cultural differences experienced within an IFDP. How participants from different countries may feel or behave when facing unfamiliar cultural values and practices in an IFDP – and how faculty developers address cultural differences – has also remained largely unexplored.

The goal of this study was to explore how different cultural values and beliefs were perceived and experienced in an IFDP. Our research questions addressed the following: (1) How was culture perceived in an IFDP? (2) What were the influences of culture on teaching and learning?

For this study, we chose the following definition of culture: “An integrated pattern of learned beliefs and behaviours that can be shared among groups and include thoughts, styles of communicating, ways of interacting, views of roles and relationships, values, practices and customs” [17]. The latter can also refer to what has been called “habits of mind”, as described in Transformative Learning Theory (TLT) [18,19]. TLT, which includes the role of critical reflection and dialectical discourse, describes how individuals transform assumptions and expectations to make their beliefs more inclusive and open to change. We used TLT to inform our data analysis and interpretation as this theory is constructivist in nature, views learning through a social lens, and has been used to study cross-cultural training, communication, and the internationalization of higher education [20,21].

## Methods

We conducted a qualitative study using interpretive description [22,23]. Interpretive description (ID) is an inductive approach “grounded in an interpretive orientation that acknowledges the constructed and contextual nature of human experience” [22]. This design allowed us to ascertain an in-depth understanding of participants’ experiences and perceptions through semi-structured interviews and translate our findings into tangible outcomes that could be applied in the context of faculty development [24]. Ethics approval was obtained from the Faculty of Medicine and Health Sciences IRB at McGill University.

### Study Setting and Sampling

The study setting was the FAIMER (Foundation for Advancement of International Medical Education and Research) Institute Fellowship, a US-based faculty development program established in 2001 that aims to improve the teaching performance and leadership skills of international health professions educators [25]. At the time of this study, this two-year program, informed by principles of transformational learning [26], was composed of two onsite sessions in Philadelphia, followed by two distance learning sessions. An education project, to be implemented in participants’ home institutions, was a focal point for learning and a vehicle for creating a transnational community of educators [27]. Almost 2000 educators from over 40 countries have participated in the FAIMER Institutes [25].

We recruited participants from five cohorts of FAIMER Fellows (2014–2019) and from FAIMER Faculty who were actively involved in the program. Criteria for active involvement included Faculty who were considered “lead faculty” in at least one session during the onsite program and Faculty who had taught in FAIMER within the last 3–5 years and were, therefore, familiar with the current program content and culture. While FAIMER offers several Regional Institutes in different countries, we selected study participants from the Philadelphia site because it represents an international cohort. To introduce the study and research team, WB and RV sent e-mails to the eligible population (72 Fellows and 10 Faculty); this was followed by a detailed e-mail invitation from SMH and YS to the same list. Altogether, 40 Fellows and 5 Faculty agreed to participate, from which we purposefully sampled participants to ensure heterogeneity in gender, nationality, disciplinary background, and cohort year. We enrolled study participants until we reached informational sufficiency, which occurred when new data did not lead to new themes or modifications of data interpretation [28].

### Data Collection and Analysis

SMH, who was not connected to the FAIMER Institute nor previously known to Fellows, conducted semi-structured interviews in the fall and winter of 2019 using Zoom or Skype to elicit Fellows’ experiences and perceptions. We pilot-tested an interview guide (Online Appendix 1), which was adjusted following the first two interviews and the investigators’ evolving conceptualizations that arose from data analysis. These changes included the order of questions asked and a shorter introduction so that we could dive into the main questions more quickly and accommodate the interview timeframe. We also modified the interview guide for FAIMER Faculty by using different wording, as needed. For example, for Faculty, we used “your teaching;” for Fellows, we used “your learning”. We also probed if FAIMER Faculty had ever changed their teaching content or process to take cultural differences into account. We deliberately avoided interview questions that could suggest discrimination, judgment, or stereotyping. Interviews lasted from 43 to 74 minutes, were audio-recorded, and were transcribed verbatim by an external professional. SMH reviewed all transcripts for accuracy.

Data analysis was iterative and started alongside data collection. We began with an inductive technique to identify preliminary patterns and common threads which were later merged to best represent the data. By using constant comparative analysis, we conducted a holistic and cross-case analysis of similar codes and themes. We also actively sought out exemplar quotations, illustrating each theme. To make sense of the findings, we gradually progressed to interpretation; this allowed us to focus on meaningful themes and elaborate on the practical application of findings. Moreover, though our data analysis was “bottom-up” as we did not generate codes using a pre-existing theoretical framework [22], we drew upon TLT as the analysis and interpretation evolved, to provide a meaningful lens on the interconnected relationship between participants and the context for teaching and learning [23].

Two authors (SMH and YS) independently read and coded the first two interviews, after which they discussed key observations and preliminary findings. SMH continued analyzing the remaining transcripts with input from YS, which led to consensus on patterns and themes. When all 15 interviews with Fellows had been coded, SMH started coding the remaining five interviews with FAIMER Faculty. SMH and YS met regularly to refine and agree on identified codes, thematic groupings, and interpretations of the findings. RV and WB reviewed the themes and gave feedback on themes, exemplar quotations, and applications of the findings.

### Reflexivity

The authors differed in their disciplinary backgrounds, educational responsibilities, and involvement in the FAIMER Institute. Two of the authors are from the US and have been involved in the FAIMER Fellowship. The two other authors are from Canada (from a French-speaking province with multiple cultures). All authors have been involved in international faculty development. Since we were asking about culture, it was important for us to discuss how we experienced and perceived cultural similarities and differences ourselves. We reflected individually and together, and we discussed how our experiences could influence the interview questions and data analysis. We were aware that our previous experiences not only shaped the questions and design of the research but might have also impacted data collection and analysis. Meeting regularly and becoming deeply engaged in the process of continuous discussion and reflection further helped us balance diverse perspectives on the interpretation of participants’ narratives.

## Results

Fifteen FAIMER Fellows and five FAIMER Faculty from 12 countries participated in the study (Table 1). Below, we present our findings according to the two areas of inquiry. Quotations are referenced by Fellows (F) or FAIMER Faculty (FF).

**Table 1:** Demographic characteristics of FAIMER Fellows and Faculty who were interviewed.


CHARACTERISTICS		FELLOWS(N = 15)	FACULTY (N = 5)

Gender	Female	8	3

	Male	7	2

Regions*	African Region	2	–

	Eastern Mediterranean Region	5	1

	European Region	1	–

	Pan American Region	2	3

	South-East Asia Region	4	1

	Western Pacific Region	1	–

Cohort Year	2014	3	

	2015	3	

	2017	5	

	2018	2	

	2019	2	

Fellowship Status	Completed	8	

	Active	7	


* To present the nationality of participants, we used the categorization developed by the World Health Organization to avoid disclosing of participants’ identities.

### 1) How was culture perceived in an IFDP?

FAIMER Fellows and Faculty commented that they perceived cultural similarities and differences in terms of specific behaviors and practices (e.g., facial expressions, body gestures, ways of addressing each other, opinion expression, physical distancing) as well as values and beliefs related to hierarchy, collaboration, documentation, and spontaneity.

*They in [X] don’t actually look at you when they talk to you. They look down, or they look over your shoulder. You are facing each other, and you are looking at them, but they are not looking at your face. (F-9)*

*One characteristic in the culture in my country, one policy they use, is top-down, from the superior to the subordinate. That’s related to the interaction between people, with junior, with senior, with the younger, with the older ones. There is a certain pattern in this country. (F-6)*

While some cultural differences were deemed “visible” in the early FAIMER encounters, some were only evident after Fellows and Faculty had spent time together. Also, several cultural differences (e.g., opinion expression) were evident when Fellows started the online sessions, whereas others (e.g., taking initiative; respecting hierarchy) were apparent when Fellows began working on their individual projects in their own countries.

*When I went back to my institution, there the culture of how to teach is quite different. People teach in isolation; they don’t want any other person to be there, so the kind of mutual collaboration between teachers was a unique aspect of FAIMER, which I am still struggling with in trying to implement it in my home institution. (F-2)*

Participants approached the notion of culture with caution, trying to avoid generalizations or oversimplification. For example, participants highlighted that another participant from their country might not share their beliefs, as people within a single country could have distinct cultural values. Participants also noted that what was perceived as a “cultural habit” might not necessarily be related to culture; instead, it could be attributed to personality, individual choice, organizational structure, or professional values.

*If you are warm and outgoing and you like to talk to people, if you are a stiff-necked person who doesn’t want to engage with people, you behave differently. But that’s in every culture. That’s personality. I don’t think I can connect it to culture. (F-9)*

“*My university is about sixty-five-years old […] the resistance to change is enormous, but I’m not going to attribute culture to this.” (F-14)*

### 2) What were the influences of cultural diversity on teaching and learning?

We identified three themes related to the influences of cultural differences on teaching and learning in an IFDP.

#### Not a barrier to learning, but a bridge to cultural awareness and network-building

Fellows believed that the cultural differences they experienced during the Institute program did not, for the most part, impede learning. They were highly motivated and passionate about making the most out of the Fellowship, and they came to the program with a fresh “mindset” that helped them understand, respect, and embrace new rules and experiences. They also valued the opportunity to question their thinking and assess some of their beliefs and assumptions.

*You can see there are some small things, but I don’t think that they would drastically affect our learning. These are just small cultural things that we have to be careful about… and I wanted to learn whatever the culture was. (F-10)*

We also noted that cultural differences enabled Fellows to achieve outcomes beyond the program objectives, including “learning about diversity” and “network building.” Many participants appreciated the existence of cultural diversity, which one Fellow described as the “beauty of life.” In addition to learning about other cultures, participants reported having an opportunity to correct false assumptions, an important aspect of critical reflection and dialectical discourse. Diversity helped participants understand that they did “not represent the world” and that what they practiced was not necessarily “the only thing or the best thing.” Additionally, exposure to different cultures helped participants with their professional development, as they felt that they had become more competent working in culturally diverse settings. One Fellow called this new insight an “eye-opener,” as before the Fellowship, she was “confined to a certain environment with a certain population sharing similar characteristics” that prevented her from “seeing other aspects of stories.”

*As I encounter different cultures and learn these rules, I feel that I’m able to be more effective each time because I remember what happened last time. So, I don’t immediately jump to my set of rules. Instead, I try to stay back, just observe, and not judge. (FF-3)*

Several participants stated that they developed strong relationships with other Fellows that continued after completing the Fellowship, and that cultural dissimilarities were not experienced as barriers to network building; rather, their description of a network of individuals with common interests and shared goals was consistent with the definition of a community of practice [29], offering opportunities for teamwork and access to resources. Network-building came as a surprise to some Fellows, as they did not expect to “build such long-lasting relationships” or “share their personal thoughts and stories” with people who were different in many aspects. One Fellow explained that she had not previously experienced building “trust at a very special level” in more homogenous settings, let alone in an international program, and she valued the opportunity to think critically about past experiences.

*We came from different environments, different levels of advancement, [different] education fields. So, we had the chance to learn from each other, to share our experiences, to collaborate. If I have some challenges at work, I just write a message asking, “Guys, I have this issue. What do you advise me?” (F-1)*

#### Unease and uncertainty leading to adaptation, modification, and mediation

The interviews revealed that certain cultural differences were experienced as subtle challenges which, at times, created uneasiness or uncertainty.

Examples of cultural behaviors that were challenging during the program, as highlighted above, included: a flat facial expression, a lack of eye contact, and holding back opinions in class, which was especially challenging for instructors who valued learners’ reactions; body gestures that might be interpreted incorrectly and lead to an “inner talk” to figure out what was happening; physical distance that might be perceived as “too intimate” or “a little frightening”; different attitudes towards time that could interrupt class function or group work; and certain religious rituals that would require program rescheduling.

*I asked her a question, and she just grabbed me and started talking in my ear. […] At that moment, I was more interested in getting away from her, instead of learning and talking. [X’s] culture is quite open, very intimate… And, I am not into hugging, especially with the opposite gender (laughs), because it’s more of a religious thing. So, probably she didn’t realize, and it was her gesture of love, but that gesture of love did not work for me. (F-10)*

Fellows also indicated some challenging cultural differences once they returned to their home countries. While almost all Fellows intended to use FAIMER’s interactive teaching methods in their own institutions, some were not able to successfully implement new initiatives. One Fellow specified that he was not sure he had “the courage to take a risk” and design an activity like “learning circles,” in which protected time was devoted to reflection and talking about non-academic personal issues to promote critical thinking and social interactions. Participants also believed that cultural practices, such as respecting hierarchy, collaboration, and documentation, could influence their projects’ success and sustainability in their home settings.

*We are very spontaneous. We have just the idea, and we are like: “let’s do it. It’s great!” (laughs). And programs are abandoned or not developed at all. Because people started in the wrong way without preparing and thinking. (F-7)*

Fellows and Faculty reported using three different approaches to address cultural dissimilarities:

*Adaptation*: Several participants observed that it was a “bit tough in the beginning” to deal with certain cultural variations, including different accents or addressing each other by first names. However, they quickly became accustomed to the new situation and found ways to adapt without much trouble.

*The fact that we had to speak to our professors or whoever they were by their first name itself was a shock for me on the first day. It took some time to adjust to that. (F-11)*

A FAIMER Faculty shared a story of the time when he started his Fellowship in FAIMER. He had decided to quit the program on the first day, because of a “little bit humiliating” experience that he described as a “cultural shock” when the Fellows were taken outside for a team-building exercise. But then, he “stayed for a few days, started to pick up the nice things in the program, and finally, was convinced about the program’s value”.

*Modification*: In most cases, participants modified their usual practices and found solutions in response to unease or uncertainty. For instance, when Fellows found it difficult to volunteer or express their opinions, FAIMER Faculty used alternative approaches, such as addressing the Fellow directly or using “parking lots”, a format in which Fellows noted their questions on a board for teachers to address anonymously later. Other examples included speaking more slowly, shifting program schedules to accommodate religious practices, and modifying educational content.

*I changed the leadership curriculum based on feedback in the first year. We were teaching fairly traditional Harvard Business School leadership, and it was not resonating with some from [X]… We adjusted, put together an international group, and redid the leadership curriculum. (FF-4)*

*Mediation*: Mediation was a less common but nonetheless effective approach in which another person acted as an advocate to bring about mutual understanding and agreement. In one situation, while two individuals involved in a conversation were not aware of the existence of a cultural disparity, a third person realized that there was a misunderstanding and stepped forward to help. In other situations, Fellows did not want to hide a perceived difference but preferred to ask someone else to advocate for them instead of getting involved directly.

*In their culture, when you are asking a question that might look like you’re questioning authority, you tell a personal story around the question to soften it. So, he told a whole story, and I didn’t understand why he was telling the story, and I didn’t understand that he was asking me a question. But there were some faculty members there who knew the culture and were able to come over to me and say,” He is worried about confronting his boss,” and I said, “Oh, yes, yes!” Then, I was able to address the question. (FF-4)*

#### Mitigation by program and professional cultures

FAIMER Faculty and Fellows noted the importance of context and observed that the influences of cultural differences on teaching and learning were mitigated by the program’s culture (i.e., values, norms, and beliefs) and participants’ common professional backgrounds and experiences as health professions educators.

*Program culture*: Fellows affirmed that they had entered a culturally sensitive program where organizers showed awareness to cultural differences by setting ground rules about respectful communications, encouraging Fellows to talk explicitly about culture, and scheduling protected time for sharing personal stories. Faculty also took advantage of forming heterogeneous groups for teamwork. Providing special places for religious rituals and making different meals available were other examples of how cultural diversity was supported. Fellows believed that their cultures were “amazingly acknowledged” and “represented” in FAIMER. Two Faculty added that FAIMER created a safe, non-judgmental environment by undertaking a continuous process of identifying and addressing cultural differences.

*One of the first things that we have is this ground rules session to agree that here we are trying to learn. These are the first things that we agree on: respect, listen, diversity is good, titles outside, and confidentiality. (FF-1)*

*Professional culture*: All FAIMER Fellows and Faculty were members of the broader community of health professions educators and shared similar interests and values as well as familiar experiences and practices. One Fellow said that it was “the most satisfying moment during the Fellowship” when he realized that the “resistance of faculty members toward change” was not a cultural problem specific to his country but could happen anywhere around the world. In diverse ways, participants identified a “common ground” related to health professions education that enabled them to minimize differences and focus on similarities reflected in their professional culture.

*Health science education is the same everywhere you go, we share the same problems: lack of faculty, lack of resources, too many students, a traditional way of teaching and learning, high interest in research. Those were shared by almost everyone. (F-14)*

## Discussion

This study explored a frequently overlooked aspect of faculty development in the health professions – the influence of culture on IFDPs – from the lens of program participants and faculty. Exploring the influences of cultural diversity on teaching and learning, we found that cultural differences were not a barrier to learning; rather, they acted as an opportunity to learn about culture and build networks. At the same time, certain cultural dissimilarities produced a sense of unease and uncertainty, which led to three constructive responses: adaptation, modification, or mediation. Context also mattered, as participants’ perspectives were affected by the program culture as well as their professional culture.

Our first theme showed that cultural differences did not impede learning. Fellows were very appreciative of the FAIMER content and variety of learner-centered approaches. They actively participated in small group discussions, completed their projects, and were inspired to apply similar methods in their home countries. This finding highlighted participants’ willingness to change perspectives and become change agents, important components of TLT [25]. It also underscored the value of critical reflection, dialectical discourse, and the ability to change in a “safe” environment. As reported above, examples of this transformative process included reflections on the behaviors of colleagues from other cultures and discussions with colleagues and faculty members familiar with that culture to better understand personal assumptions and behaviors. While several previous studies have reported that specific cultural aspects, like obedience to hierarchy and respect for seniority, influenced participants’ learning (and at times a tendency to prefer a more traditional lecture-dominated program) [30,31,32], our findings are consistent with reports on an established US-based IFDP which was delivered across a variety of countries [33,34,35,36], with successful results. Our findings might also be explained by expectancy-value theory, which links achievement performance to individuals’ expectancy-related and task-value beliefs [37]. It is likely that in our study, Fellows were “expecting valuable outcomes” from a prestigious American program with a strong reputation. It is also possible that Fellows believed that this program would contribute to their professional growth, which not only resonated with their internal beliefs about self-improvement (intrinsic value) but would also increase the probability of external benefits such as potential career advancement in their home countries (utility value). In diverse ways, Fellows’ motivation to learn may have overridden challenges related to perceived cultural disparities.

As noted in our second theme, some FAIMER Fellows experienced unease and uncertainty due to certain cultural differences, primarily those emanating from different interpersonal communication styles. A similar finding was reported when a US-based program, transferred to another country, encountered challenges resulting from participants’ emotional self-control, silence, and avoidance of direct eye contact [13]. Our study adds to this evidence by identifying three constructive responses (adaptation, modification, and mediation) to these possible challenges. While adaptation was mentioned more frequently by Fellows, modification was used more by FAIMER Faculty, and mediation by a third person was identified as an effective strategy for both Fellows and Faculty to resolve tensions or avoid cultural confrontation. TLT [25], which has been shown to have utility in describing how people develop global awareness and competence [21], asserts that when an individual is involved in a situation that is unfamiliar to them, they will either reject it or, in most cases, adapt, as seen in our study. Critical reflection and dialectical discourse also helped this transformative process.

We also noted that our findings were situated in a specific program and professional culture. FAIMER is informed by principles of TLT and characterized by a carefully designed curriculum, a culturally sensitive environment, responsive Faculty, and highly motivated learners. It is likely that these (and other) efforts contributed to some of our findings, enabling transformative learning as well as network building and solution-oriented responses toward cultural differences. Furthermore, since our participants were all health professions educators, their professional culture might have influenced how they perceived and addressed cultural differences. We found, and other studies have also reported, commonalities among health professions educators in terms of disciplinary backgrounds, experiences, values, beliefs, and needs [3,33,35]. As many universities are conducting curricular reforms and moving toward student-centered methods [16,35], and global standards and accreditation processes are increasingly recognized and accepted [38], it is not surprising that principles of instructional design and delivery are generally well-received [16,38]. Participants’ professional backgrounds and desires to seek “common ground” and commit to action, together with the program’s emphasis on critical reflection and dialectical discourse, may have helped to transcend cultural differences [18], which in turn, can lead to positive future action and meaningful relationships [19].

### Strengths and Limitations

We chose the FAIMER Fellowship because it offered a multicultural environment with Fellows and Faculty from different countries and was composed of onsite and online training within a longitudinal program, thus enabling the exploration of diverse instructional methods. As we interviewed FAIMER Faculty, current Fellows, and Fellows who had previously completed their Fellowship, we were able to triangulate our data sources and enhance trustworthiness. However, this study investigated only one IFDP, and participation was voluntary; hence, findings might not be transferable to other settings. Recall bias was another challenge, since we asked participants to recall experiences that had occurred in the past. Lastly, some aspects of teaching and learning might have been better understood by observing interactions during the Fellowship.

### Implications for Education and Research

The findings of this study advance our understanding of the influence of culture on IFDPs and enable us to make suggestions for enhancing faculty development activities and recommending future research directions.

We have summarized the educational implications for faculty developers and participants in IFDPs in Table 2. From a research perspective, we encourage educators to examine IFDPs in different locations; for example, FAIMER Regional Institutes could be a valuable data source for exploring the influence of culture in “home” settings. Additionally, using other methods such as ethnography could provide more insights into the influence of culture on teaching and learning in IFDPs, with a focus on the influence – and interaction – of professional and program cultures as well. Further exploration of faculty development through the lens of TLT would also be worthwhile.

**Table 2:** Educational implications for IFDP developers, faculty developers, and participants.


	GOAL	STRATEGIES

Program developers	To foster respect for other cultures To facilitate explicit communication around culture To facilitate network building To develop cultural competence	Planning meals for different diets, providing facilities for prayer, and considering religious practices when scheduling Establishing and communicating specific ground rules Talking explicitly about cultural differences, their influences, and the common responses toward them (adaptation, modification, and mediation) Devoting specific time to share personal stories (like “learning circles”) Forming heterogeneous small groups

Faculty developers	To experience cultural diversity To respond effectively to cultural differences	Visiting other countries Being aware of different interpersonal communications and working styles in different cultures Speaking slowly and clearly, addressing participants directly, asking individual questions, using “parking lots” Asking an experienced person to “mediate” and help with “decoding” cultural differences

Participants	To get prepared for a new situation To respond effectively to cultural differences To implement educational projects successfully	Developing a fresh mindset Being aware of different interpersonal communications and working styles in different cultures Asking an experienced person to “mediate” Considering cultural differences in home countries (hierarchy, collaboration, documentation, and spontaneity)


## Conclusion

The cultural diversity of health professions educators in an IFDP does not seem to be a barrier to learning. Certain perceived cultural differences may cause subtle challenges, but they are often managed constructively to avoid tension. Program culture and professional culture may, at times, override cultural beliefs, values and norms.

*We have that same human nature, but then we adapt according to the culture. We wrap it around ourselves, and then we try to act accordingly what is most suitable in that specific culture. There are so many similarities, the concept of human nature, the concept of healer, the concept of teacher. These are some of the universal things. (F-10)*

## Additional File

The additional file for this article can be found as follows:

10.5334/pme.31.s1 Online Appendix. Sample questions in the interview guide.
