# Supplementary material for: Understanding and Embracing Culture in International Faculty Development
Source: Perspect Med Educ. 2023 Jan 4;12(1):1–11. doi: 10.5334/pme.31 (PMC9997115; doi:10.5334/pme.31)
Supplement: Online Appendix. — Sample questions in the interview guide. [file pme-12-1-31-s1.pdf]

Online Appendix: Sample questions in the interview guide.

Categories	Sample Questions
Participants' experiences with the FAIMER Fellowship	• Could you describe one of the most satisfying/challenging moments about the Fellowship?<br>• Was there anything in the Fellowship that surprised you, something that you did not expect, or that you were not used to?
Perceived cultural differences	• How do you compare teaching and learning in FAIMER with the teaching and learning in your country/other settings?<br>• Have you ever experienced a cultural difference during FAIMER Fellowship? Please specify.<br>• What was your reaction?
The role of culture in faculty development	• How, if at all, do you think culture plays a role in teaching and learning?<br>• How do you think that cultural differences influenced you/your learning/ your involvement/your teaching?<br>• Do you think that the FAIMER content or process could be modified to take cultural differences into account? Please specify.<br>• Do you think Fellows should be prepared in a particular way before participating in FAIMER?
The influence of culture on teaching, learning, and scholarship in home countries	• How do you compare teaching and learning methods of FAIMER with the teaching and learning methods in your country?<br>• Did you have any challenges and difficulties when you were implementing your project in your home setting? Could any of these challenges be attributed to cultural issues?
